# Biomarker response to high-specific-activity I-131 meta-iodobenzylguanidine in pheochromocytoma/paraganglioma

**DOI:** 10.1530/ERC-22-0236

**Published:** 2022-01-05

**Authors:** Camilo Jimenez, Bennett B Chin, Richard B Noto, Joseph S Dillon, Lilja Solnes, Nancy Stambler, Vincent A DiPippo, Daniel A Pryma

**Affiliations:** 1 University of Texas MD Anderson Cancer Center, Houston, Texas, USA; 2 University of Colorado Anschutz Medical Campus, Aurora, Colorado, USA; 3 Warren Alpert Medical School of Brown University, Providence, Rhode Island, USA; 4 University of Iowa, Iowa City, Iowa, USA; 5 Johns Hopkins Medicine, Baltimore, Maryland, USA; 6 Progenics Pharmaceuticals, Inc., a Lantheus Company, North Billerica, Massachusetts, USA; 7 Perelman School of Medicine at the University of Pennsylvania, Philadelphia, Pennsylvania, USA

**Keywords:** high-specific-activity I-131 MIBG, biomarker response, pheochromocytoma, paraganglioma

## Abstract

The objective of this study is to present the complete biomarker response dataset from a pivotal trial evaluating the efficacy and safety of high-specific-activity I-131 meta-iodobenzylguanidine in patients with advanced pheochromocytoma or paraganglioma. Biomarker status was assessed and post-treatment responses were analyzed for catecholamines, metanephrines, and serum chromogranin A. Complete biomarker response (normalization) or partial response, defined as at least 50% reduction from baseline if above the normal range, was evaluated at specified time points over a 12-month period. These results were correlated with two other study objectives: blood pressure control and objective tumor response as per RECIST 1.0. In this open-label, single-arm study, 68 patients received at least one therapeutic dose (~18.5 GBq (~500 mCi)) of high-specific-activity I-131 meta-iodobenzylguanidine. Of the patients, 79% and 72% had tumors associated with elevated total plasma free metanephrines and serum chromogranin A levels, respectively. Best overall biomarker responses (complete or partial response) for total plasma free metanephrines and chromogranin A were observed in 69% (37/54) and 80% (39/49) of patients, respectively. The best response for individual biomarkers was observed 6–12 months following the first administration of high-specific-activity I-131 meta-iodobenzylguanidine. Biochemical tumor marker response was significantly associated with both reduction in antihypertensive medication use (correlation coefficient 0.35; *P* = 0.006) as well as objective tumor response (correlation coefficient 0.36; *P* = 0.007). Treatment with high-specific-activity I-131 meta-iodobenzylguanidine resulted in long-lasting biomarker responses in patients with advanced pheochromocytoma or paraganglioma that correlated with blood pressure control and objective response rate. ClinicalTrials.gov number: NCT00874614.

## Introduction

Pheochromocytomas and paragangliomas (PPGLs) are rare neuroendocrine tumors derived from chromaffin cells of the adrenal medulla and paraganglia, respectively (Jimenez 2018). Each year, approximately 500–1600 new cases of PPGLs are diagnosed in the United States (Grogan *et al.* 2011, Hamidi *et al.* 2017). Although most PPGLs are localized and surgical resection is the standard first-line intervention, locally invasive or metastatic disease is estimated to occur in 10–35% of cases (Amar *et al.* 2005, Grogan *et al.* 2011, Kimura *et al.* 2014, Hamidi *et al.* 2017, Lam 2017, Wakabayashi *et al.* 2019, Fishbein *et al.* 2021). Patients with metastatic or unresectable PPGLs have high morbidity and mortality rates owing to disease progression and/or cardiovascular sequelae (Feng *et al.* 2011, Ayala-Ramirez *et al.* 2012, Roman-Gonzalez *et al.* 2018).

PPGLs usually hypersecrete catecholamines, which are responsible for many of the observed signs and symptoms of disease, including paroxysmal hypertension, palpitations, anxiety, headaches, and constipation (van Berkel *et al.* 2014, Thosani *et al.* 2015, Falhammar *et al.* 2018). Patients with these tumors are prone to developing catecholamine-mediated crises characterized by hypertensive emergencies and cardiovascular events (Zelinka *et al.* 2012). Elevated urinary fractionated and plasma free metanephrines have been shown to be sensitive diagnostic markers of PPGL (Sawka *et al.* 2003, Lenders *et al.* 2014, Corcuff *et al.* 2017). In addition, patients with metastatic PPGLs have been reported to have higher metanephrine levels than those with localized PPGLs (Feng *et al.* 2011). Although less sensitive in the diagnostic setting, chromogranin A (CgA) levels may also be valuable in following response to therapy and monitoring for recurrence (Parisien-La Salle *et al.* 2021). Annual biochemical follow-up is typically life-long for patients at high risk for recurrent or metastatic disease (i.e. those with paraganglioma, diagnosis at a young age, multiple or large primary tumors, or genetic mutations) (Ayala-Ramirez *et al.* 2011, Plouin *et al.* 2016).

High-specific-activity (HSA) I-131 meta-iodobenzylguanidine (MIBG) is the first and only US Food and Drug Administration (FDA)-approved treatment for patients aged 12 years and older with iobenguane-scan positive, unresectable, locally advanced or metastatic PPGL who require systemic anticancer therapy (AZEDRA® 2021). The unique synthesis and manufacturing of HSA I-131 MIBG permits an administered dose that consists almost entirely of I-131–labeled MIBG (Vaidyanathan & Zalutsky 1993, Coleman *et al.* 2009, Barrett *et al.* 2010, Vallabhajosula & Nikolopoulou 2011, Noto *et al.* 2018, Pryma *et al.* 2019). Approval of the drug was supported by the results of a pivotal phase 2 clinical trial demonstrating improvement of blood pressure control in the majority of patients being treated for hypertension and a high clinical benefit rate (>90%) including partial responses noted in individuals treated with two doses (~37 GBq; (~1000 mCi)) (Pryma *et al.* 2019). Prior treatment paradigms for unresectable PPGL included the research and compassionate use of conventional, low-specific-activity I-131 MIBG and cytotoxic chemotherapy with cyclophosphamide, vincristine, and dacarbazine (Loh *et al.* 1997, Gonias *et al.* 2009, Plouin *et al.* 2012, Niemeijer *et al.* 2014, van Hulsteijn *et al.* 2014, Jimenez *et al.* 2019).

Given that most PPGLs secrete catecholamines and the clinical presentation is frequently related to their secretory products, it is reasonable to postulate a correlation between treatment efficacy and biomarker response. A retrospective analysis evaluating 16 studies reported complete biochemical response (CR) rates of 0–27%, 16–100% partial response (PR), and 0–63% stable disease (SD) with low-specific-activity I-131 MIBG therapy (van Hulsteijn *et al.* 2014). In a phase 1 study of HSA I-131 MIBG for 21 patients with metastatic or recurrent PPGL, best biochemical response (CR or PR) rates of 80% for serum CgA and 64% for total metanephrines were observed (Noto *et al.* 2018).

Previously, as the biochemical response was a secondary endpoint and not the focus of our pivotal clinical study, we only described limited topline data for select biomarkers at a single 12-month time point (Pryma *et al.* 2019). Here, we report for the first time, best biochemical responses at any time point for serum CgA and total plasma free and urinary metanephrines, plasma free and urinary normetanephrine, and plasma free and urinary metanephrine (including waterfall plots for all patients), longitudinal biomarker responses for the study duration (0 to 12 months including 3-, 6-, and 9-month time points), the extent of biochemically SD and progressive disease (PD), and significant correlations with objective responses and the primary endpoint for the pivotal trial with HSA I-131 MIBG in patients with metastatic or recurrent, unresectable PPGL.

## Subjects and methods

### Trial design, patients, and treatment

A multicenter, open-label, single-arm trial was conducted under a Special Protocol Assessment agreement with the FDA. The primary efficacy endpoint was the proportion of evaluable patients with at least a 50% reduction of all baseline antihypertensive medications lasting for at least 6 months beginning in the first 12 months following the first therapeutic dose. Key secondary endpoints included the evaluation of radiographic tumor and biochemical tumor marker responses. Patients were enrolled at 10 centers in the United States and followed for 12 months for efficacy and up to 5 years for long-term safety. Baseline demographics and concomitant medications were recorded.

Eligible patients were aged 12 years or older with a confirmed diagnosis of metastatic or unresectable PPGL and the ability to provide written informed consent. Patients included in the study were ineligible for curative surgery and had received prior therapy for PPGL that had failed or were not candidates for chemotherapy or other curative therapies at study entry. All patients were on a stable antihypertensive medication regimen for at least 30 days prior to the first therapeutic dose, had at least one tumor site identified by CT or MRI, and had definitive MIBG avidity. Key exclusion criteria were a platelet count of <80,000/µL, an absolute neutrophil count of <1200/µL, and a creatinine clearance of <30 mL/min.

Patients underwent treatment planning by receiving ~185 MBq (5 mCi) of the drug, followed by serial whole-body scans to assess MIBG avidity and biodistribution and to conduct dosimetry calculations to determine radiation absorbed dose to normal organs (Noto *et al.* 2018, Jimenez *et al.* 2019, Pryma *et al.* 2019). Patients who showed MIBG tumor avidity received up to two therapeutic doses of the drug, each planned at ~18.5 GBq (500 mCi) or 296 MBq/kg (8 mCi/kg) for patients weighing ≤62.5 kg, administered intravenously approximately 90 days apart. Individualized dose reduction was undertaken to ensure that radiation-absorbed doses to critical organs would not exceed published toxicity limits after two therapeutic doses (Barrett *et al.* 2010). To receive the second therapeutic dose of the drug, patients’ hematologic values were required to return to baseline levels or within the normal range within 24 weeks following the first therapeutic dose. Patients who did not receive the second therapeutic dose were requested to be followed for study assessments including biomarkers.

### Efficacy assessments

As previously described (Pryma *et al.* 2019), blood pressure response, the study’s primary endpoint, was measured by determining whether a patient had at least a 50% reduction of all baseline antihypertensive medications for a minimum of 6 months. Radiographic tumor response was assessed by the Response Evaluation Criteria in Solid Tumors (RECIST) version 1.0 (Therasse *et al.* 2000). Each patient underwent a baseline CT/MRI study of the chest, abdomen, and pelvis and had follow-up tumor imaging every 3 months during the 12-month efficacy period. Patients whose CT/MRI study showed PR or CR underwent a follow-up CT/MRI study for confirmation. Biochemical tumor markers associated with PPGL (serum CgA, plasma and 24-h urinary catecholamines (norepinephrine, epinephrine)) and plasma free and urinary fractionated metanephrines (metanephrine, normetanephrine) were measured for evaluation of response. Tumor marker samples were collected as per study protocol laboratory manual guidelines (ACM Global Central Laboratory; Rochester, NY, USA) at multiple predefined time points for all treated patients and assessed by a central laboratory. Brief descriptions of these proprietary biochemical assays are provided later. Since this study was initiated in 2009, methoxytyramine was not evaluated. As prespecified in the study analysis plan, patients with hypersecretory tumors with any biomarker ≥1.5 times the upper limit of normal (ULN) at baseline were included in this data analysis. This cut-off was chosen in order to include patients with oligometastatic unresectable disease in whom plasma metanephrines and normetanephrines are not higher than 4× ULN, thus allowing for correlation analysis with the primary and secondary endpoints. Biochemical CR (normalization), PR (>50% decrease from baseline value but remaining above ULN), SD (≤50% decrease from baseline value or increase by ≤50% of baseline value, and remaining above the ULN), and PD (>50% increase from baseline value) were confirmed at the next assessment. Following treatment, tumor markers were measured every 2 weeks during weeks 2–24 and monthly during months 7–12 following the first therapeutic HSA I-131 MIBG infusion. The biochemical response was defined as biochemical CR and PR; best responses were determined at any time point after the first treatment dose. To assess the impact of the concomitant use of proton pump inhibitors (PPIs) on CgA response, these patients were identified for additional analyses.

### Biomarker assays

All proprietary biochemical assays for this analysis were performed by a central laboratory. CgA levels were determined using a complement-enzyme-linked immunosorbent assay. Plasma and 24-h urinary catecholamines were determined by HPLC. Plasma free and urinary fractionated metanephrine and normetanephrine levels were determined using a liquid chromatography and tandem mass spectrometry system.

### Statistical analyses

Patients included in this analysis received at least one therapeutic dose of the drug. No imputation for missing values, other than partial dates, was performed. Descriptive statistics are presented. For continuous measures, the mean, standard deviation, median, range, and sample size were calculated. For categorical measures, the number of patients and respective percentages in each category were determined. Pearson correlations between numerically transformed biochemical tumor marker responses and either (i) the primary endpoint or (ii) objective tumor response by RECIST 1.0 were computed and presented as appropriate. Fisher’s exact *P*-values for the categorical response tabulations were also calculated and presented. All statistical analyses and data listings were produced using SAS software version 9.4 or JMP software version 14.2 (SAS Institute, Cary, NC, USA).

### Trial oversight

Each study center’s Institutional Review Board (IRB) approved the study protocol and all amendments. Those IRBs include the Duke University Health System Institutional Review Board for Clinical Investigations; The University of Texas M.D. Anderson Cancer Center Office of Protocol Research; the Rhode Island Hospital IRB; the Western Institutional Review Board (WIRB); the University of Pennsylvania Insitutional Review Board; and the John Hopkins Medicine Office of Human Subjects Research Institutional Review Board #1.

A written informed consent form was signed by all patients (or, for patients younger than 18 years, legal guardians). This study was performed in accordance with the Declaration of Helsinki, the International Conference on Harmonisation Good Clinical Practice guidelines, and all applicable regulations. An independent data monitoring committee was established and utilized to safeguard study integrity and assess the safety and efficacy of the interventions.

## Results

**Figure 1:**
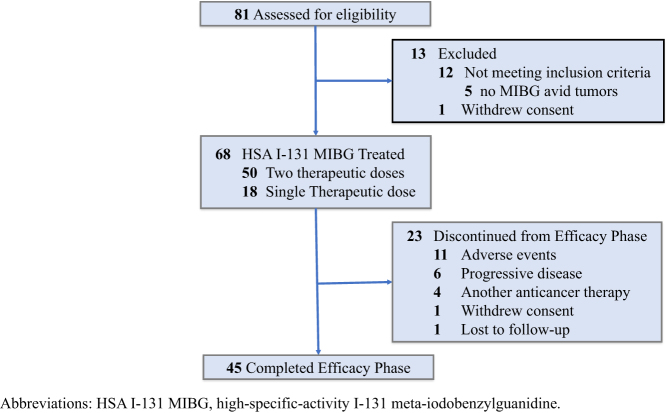
Patient flow diagram for the phase 2 trial.

The patient flow for this trial is summarized in Fig. 1. Sixty-eight patients received at least one dose of HSA I-131 MIBG. Select baseline clinical characteristics are presented in Table 1.

**Table 1:** Select demographics and baseline characteristics of 68 patients with advanced pheochromocytoma or paraganglioma who received at least 1 therapeutic dose of high-specific-activity I-131 meta-iodobenzylguanidine (HSA I-131 MIBG). All data are no. of patients (%) unless otherwise indicated.

Characteristics	No. of patients (%)
Sex	
Male	39 (57.4)
Female	29 (42.6)
Age, years	
Mean (s.d.)	50.9 (13.9)
Median (range)	54.5 (16–72)
<18	1 (1.5)
18–30	7 (10.3)
31–64	49 (72.1)
>64	11 (16.2)
Primary diagnosis	
Pheochromocytoma	53 (77.9)
Paraganglioma	14 (20.6)
Pheochromocytoma + paraganglioma	1 (1.5)
Baseline elevated tumor marker status	
Norepinephrine	32 (47.1)
Mixed catecholamines	24 (35.3)
Epinephrine	4 (5.9)
None	6 (8.8)
No data available	2 (2.9)
Prior MIBG treatments	
Yes	21 (30.9)
No	47 (69.1)
Location of metastases^a^	
Lung and/or liver metastases	32 (50)
No lung or liver metastases	32 (50)
Bone metastases	
Yes	39 (57.4)
No	29 (42.6)

^a^Data provided for 64 patients with evaluable target lesions at baseline.

### Best overall (complete response + partial response) biochemical tumor marker response

**Figure 2:**
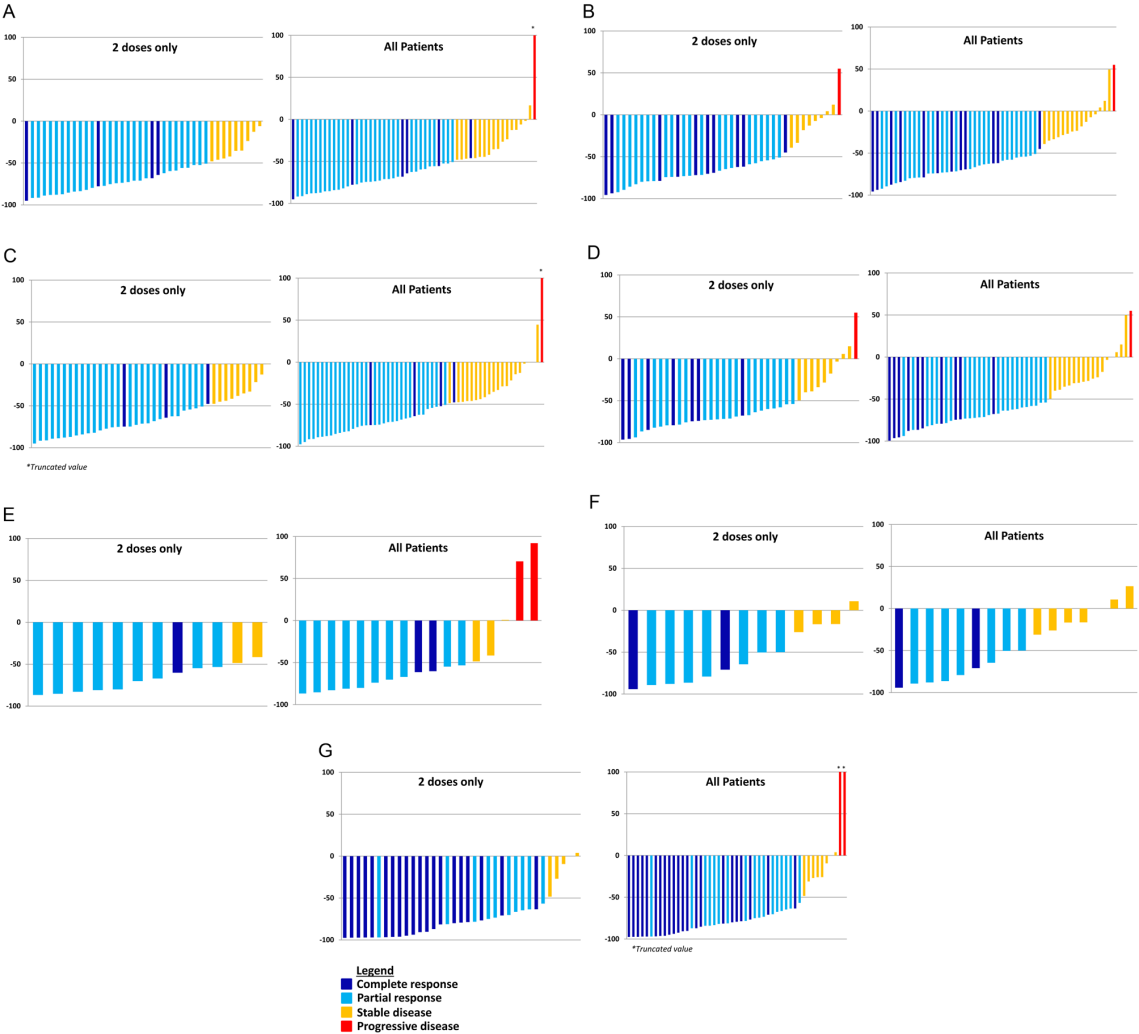
Best biochemical tumor marker response at any time point for (A) total plasma free metanephrines (normetanephrine + metanephrine); (B) total fractionated urinary metanephrines (normetanephrine + metanephrine); (C) plasma free normetanephrine; (D) urinary normetanephrine; (E) plasma free metanephrine; (F) urinary metanephrine; (G) chromogranin A.

**Figure 3:**
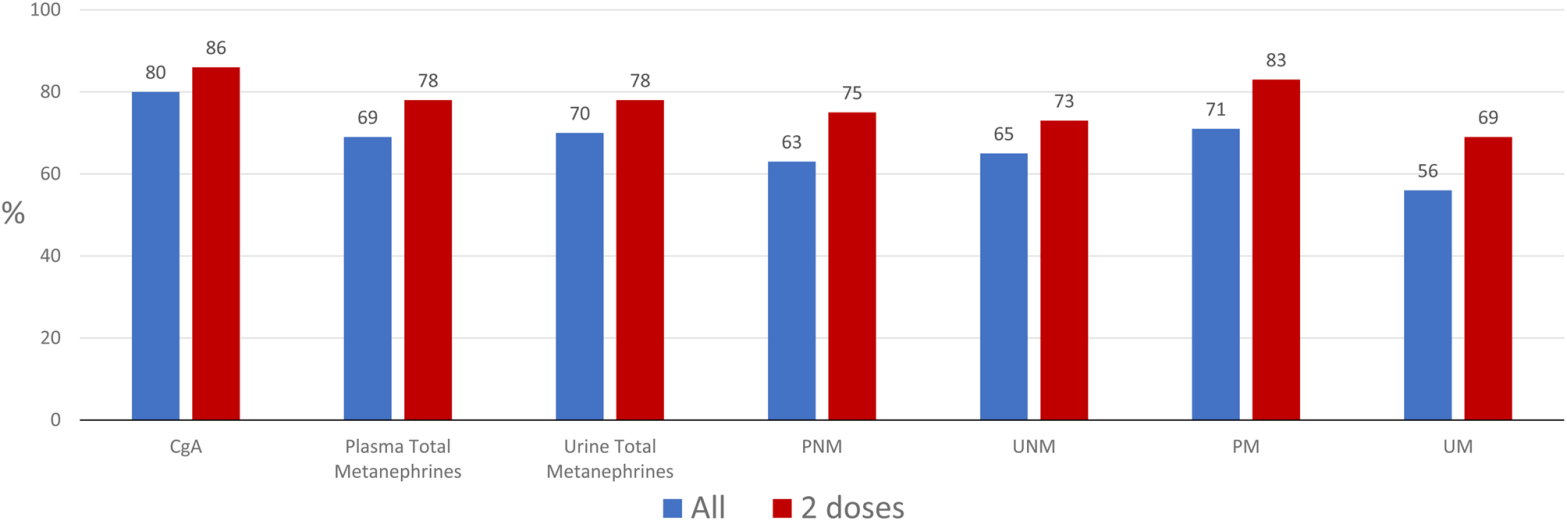
Proportion of patients with a biochemical response (CR + PR) at any time point for all patients receiving a therapeutic dose of HSA I-131 MIBG (blue bars) compared to patients who received two doses (red bars). PNM: plasma normetanephrine; UM: urinary metanephrine; PM: plasma metanephrine; UM: urinary normetanephrine.

Fifty-four (79%) patients had high plasma total metanephrines, 53 (78%) had high urinary fractionated metanephrines, and 49 (72%) patients had elevated serum CgA levels. The proportions of patients achieving a biochemical response (CR or PR) in plasma analyses at any time after treatment with at least one dose of HSA I-131 MIBG were as follows: total free metanephrines 69%, free metanephrine 71%, and free normetanephrine 63%. Corresponding proportions for urinary analyses were as follows: total metanephrines 70%, metanephrine 56%, and normetanephrine 65%. Eighty percent of patients achieved a biochemical response (CR or PR) for serum CgA. (Table 2 and Fig. 2A, B, C, D, E, F and G). The best biochemical response rates in only patients who received two therapeutic doses of HSA I-131 MIBG were higher when compared to all treated patients (Fig. 3).

**Table 2:** Best overall biomarker response in patients with elevated baseline biomarkers.

	Chromogranin A		Plasma total metanephrines		Urine total metanephrines		Plasma normetanephrine		Urine normetanephrine		Plasma metanephrine		Urine metanephrine	
	No.	%	No.	%	No.	%	No.	%	No.	%	No.	%	No.	%
**Patients receiving any therapeutic dose of HSA I-131 MIBG**														
Baseline 1.5× upper limit of normal	49	-	54	-	53	-	56	-	52	-	17	-	16	-
Complete response + partial responses	39	80	37	69	37	70	35	63	34	65	12	71	9	56
Complete response	22	45	6	11	12	23	4	7	10	19	2	12	2	13
Partial response	17	35	31	57	25	47	31	55	24	46	10	59	7	44
Stable disease	8	16	16	30	15	28	20	36	17	33	3	18	7	44
Progressive disease	2	4	1	2	1	2	1	2	1	2	2	12	0	0
**Patients receiving two therapeutic doses of HSA I-131 MIBG**														
Baseline 1.5× upper limit of normal	35	-	40	-	40	-	40	-	38		12	-	13	-
Complete response + partial responses	30	86	31	78	31	78	30	75	28	74	10	83	9	69
Complete response	20	57	4	10	10	25	3	8	7	18	1	8	2	15
Partial response	10	29	27	68	21	53	27	68	21	55	9	75	7	54
Stable disease	5	14	9	23	8	20	10	25	9	24	2	17	4	31
Progressive disease	0	0	0	0	1	3	0	0	1	3	0	0	0	0

HSA I-131 MIBG, high-specific-activity I-131 meta-iodobenzylguanidine.

### Biomarker responses over time

**Figure 4:**
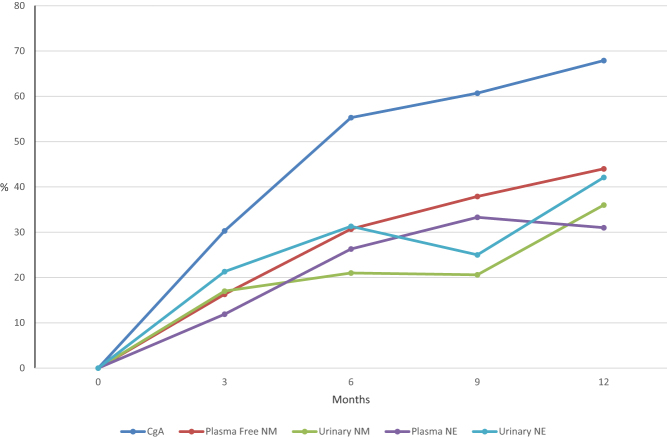
Individual biochemical tumor marker responses: percentage of patient responders (complete response plus partial response) over time. CgA: chromogranin A; NM: normetanephrines; NE: norepinephrine.

As indicated in Table 1, the majority of patients with metastatic or unresectable tumors exclusively or predominantly secrete norepinephrine. Therefore, to further assess catecholaminergic biomarker responses, norepinephrine and normetanephrines were evaluated over time. The number and proportion of patients who had confirmed biochemical PR or CR following treatment increased over time. For serum CgA (*n* = 49), plasma (*n* = 52) and urinary (*n* = 41) norepinephrine, and plasma free (*n* = 57) and urinary (*n* = 53) normetanephrine, responses were observed in at least 10% of evaluable patients as early as 3 months from the first dose (Fig. 4). Responses of specific biomarkers to HSA I-131 MIBG are described as follows:

#### Serum chromogranin A

The percentage of serum CgA-related tumor marker responders (CR + PR) steadily increased in the 12 months following the first therapeutic dose reaching 68% (19/28) (Fig. 4). Only 4 of 28 patients (14%) were confirmed to have PD at the 12-month time point (Table 3).

**Table 3:** Biomarker response rates over time.

Biomarker	No. (%)			
	Month 3	Month 6	Month 9	Month 12
Serum chromogranin A				
No. of patients	43	38	28	28
Complete + partial responses	13 (30)	21 (55)	17 (61)	19 (68)
Complete response	3 (7)	8 (21)	9 (32)	8 (29)
Partial response	10 (23)	13 (34)	8 (29)	11 (39)
Stable disease	28 (65)	16 (42)	10 (36)	5 (18)
Progressive disease	2 (5)	1 (3)	1 (4)	4 (14)
Plasma free normetanephrine				
No. of patients	43	39	29	25
Complete + partial responses	7 (16)	12 (31)	11 (38)	11 (44)
Complete response	1 (2)	2 (5)	2 (7)	0 (0)
Partial response	6 (14)	10 (26)	9 (31)	11 (44)
Stable disease	35 (81)	25 (64)	17 (59)	13 (52)
Progressive disease	1 (2.3)	2 (5)	1 (3)	1 (4)
Urinary normetanephrine				
No. of patients	41	38	29	25
Complete + partial responses	7 (17)	8 (21)	6 (21)	9 (36)
Complete response	1 (2)	1 (3)	1 (3)	1 (4)
Partial response	6 (15)	7 (18)	5 (17)	8 (32)
Stable disease	32 (78)	26 (68)	20 (69)	13 (52)
Progressive disease	2 (5)	4 (11)	3 (10)	3 (12)
Plasma norepinephrine				
No. of patients	42	38	30	29
Complete + partial responses	5 (12)	10 (26)	10 (33)	9 (31)
Complete response	0 (0)	0 (0)	0 (0)	1 (3)
Partial response	5 (12)	10 (26)	10 (33)	8 (28)
Stable disease	37 (88)	27 (71)	20 (67)	19 (66)
Progressive disease	0 (0)	1 (3)	0 (0)	1 (3)
Urinary norepinephrine				
No. of patients	33	32	24	19
Complete + partial responses	7 (21)	10 (32)	6 (25)	8 (42)
Complete response	2 (6)	4 (13)	4 (17)	3 (16)
Partial response	5 (15)	6 (19)	2 (8)	5 (26)
Stable disease	22 (67)	19 (59)	15 (63)	9 (47)
Progressive disease	4 (12)	3 (9)	3 (13)	2 (11)

Thirty-five of 68 (51.5%) patients received concomitant PPI treatment. Of the 49 patients with elevated baseline CgA, 27 (55.1%) were receiving PPIs. The best CgA response for the 27 patients receiving PPIs was 21 (78%) CR/PR, 4 (15%) SD, and 2 (7%) PD. The best CgA response for the 22 patients that did not receive PPIs was 18 (82%) CR/PR, 4 (18%) SD, and 0 (0%) PD.

#### Plasma and urinary norepinephrine

Plasma norepinephrine-related tumor marker response (CR + PR) stabilized in the 9–12 months following the first therapeutic dose with 33% (10/30) of patients responding at the 9-month time point and only 1 of 29 patients (3%) confirmed to have PD at the 12-month point (Fig. 4 and Table 3). The percentage of urinary norepinephrine-related tumor marker responders (CR + PR) increased in the 12 months following the first therapeutic dose reaching 42% (8/19) (Fig. 4). Only 2 of 19 patients (10.5%) were confirmed to have PD at the 12-month point (Table 3).

#### Plasma free and urinary normetanephrine

The percentage of plasma free normetanephrine-related tumor marker responders (CR + PR) steadily increased in the 12 months following the first therapeutic dose (Fig. 4) with 44% (11/25) responding and only one of 25 patients (4%) was confirmed to have PD at the 12-month point (Table 3). The percentage of urinary normetanephrine-related tumor marker responders (CR + PR) also steadily increased in the 12 months following the first therapeutic dose reaching a 36% (9/25) response rate (Fig. 4). Three of 25 patients (12%) were confirmed to have a >50% increase in urinary normetanephrine levels (PD) at the 12-month point (Table 3).

### Correlation of biomarker response rates to primary endpoint and objective response rates

Overall biomarker responses correlated weakly but significantly with responder status for the primary outcome assessment (i.e. >50% reduction in antihypertensive medications for at least 6 months; Table 4). For all patients with hypersecretory tumors (with a baseline biochemical marker level of ≥1.5× ULN for all tested biomarkers), a comparison of biomarker response with a reduction in antihypertensive therapy yielded a correlation coefficient of 0.35 (*P* = 0.006). For patients with norepinephrine-only-hypersecreting tumors, a comparison of biomarker response with antihypertensive therapy yielded a correlation coefficient of 0.47 (*P* = 0.008). For patients with norepinephrine and epinephrine-hypersecreting tumors, a comparison of biomarker response with antihypertensive therapy yielded a correlation coefficient of 0.37 (*P* = 0.006).

**Table 4:** Correlation of overall tumor biomarker response with the primary endpoint.^a^

	Overall tumor biomarker response	Primary efficacy outcome^a^, *n* (%)		Correlation coefficient (*P*-value)
		Responder	Non-responder	Fisher’s exact *P*-value
All baseline ≥ 1.5x ULN (*n* = 60)	Responder	7 (12)	6 (10)	0.35 (0.006) 0.012
	Non-responder	8 (13)	39 (65)	
NE-hypersecreting (*n* = 56)	Responder	7 (12.5)	6 (11)	0.37 (0.006) 0.011
	Non-responder	7 (12.5)	36 (64)	
NE-only-hypersecreting (*n* = 31)	Responder	6 (19)	4 (13)	0.47 (0.008) 0.015
	Non-responder	3 (10)	18 (58)	
EPI-only-hypersecreting (*n* = 4)	Responder	0 (0)	0 (0)	N/A
	Non-responder	1 (25)	3 (75)	

^a^Primary efficacy outcome = reduced antihypertensive medications by ≥50% for at least 6 months.

EPI, epinephrine; NE, norepinephrine; ULN, upper limit of normal.

The overall biomarker response also weakly but significantly correlated with objective tumor response (best confirmed response of CR or PR based on RECIST 1.0 criteria; Table 5). For all patients with hypersecretory tumors, a comparison of biomarker response with objective tumor response yielded a correlation coefficient of 0.36 (*P* = 0.007).

**Table 5:** Correlation of overall tumor biomarker response with objective tumor response.^a^

	Overall tumor biomarker response	RECIST response^a^, *n* (%)			Correlation coefficient (*P*-value)
		PR	SD	PD	Fisher’s exact *P*-value
All baseline ≥ 1.5x ULN (*n* - =55)	Responder	7 (13)	6 (11)	0 (0)	0.36 (0.007) 0.012
	Non-responder	7 (13)	32 (58)	3 (5)	
NE-hypersecreting (*n* = 52)	Responder	7 (13)	6 (12)	0 (0)	0.38 (0.005) 0.010
	Non-responder	6 (12)	30 (58)	3 (6)	
NE-only-hypersecreting (*n* = 28)	Responder	5 (18)	5 (18)	0 (0)	0.35 (0.065) 0.091
	Non-responder	3 (11)	13 (46)	2 (7)	
EPI-only-hypersecreting (*n* = 3)	Responder	0 (0)	0 (0)	0 (0)	N/A
	Non-responder	1 (33)	2 (67)	0 (0)	

^a^Best confirmed response of complete response or partial response according to Response Evaluation Criteria in Solid Tumors.

EPI, epinephrine; NE, norepinephrine; PD, progressive disease; PR, partial response; SD, stable disease; ULN, upper limit of normal.

## Discussion

These results indicate that treatment with HSA I-131 MIBG substantially decreases the excessive secretion of catecholamines in patients with metastatic PPGLs. These hormonal reductions are long-lasting, improve over time, and correlate with blood pressure control and oncological responses.

This is one of the largest prospective clinical trials to date in patients with advanced PPGL and has demonstrated multiple clinical benefits of HSA I-131 MIBG. Although several patients did not achieve the blood pressure reduction benefit as defined in the primary endpoint, most had a reduction in the number and doses of antihypertensives (Pryma *et al.* 2019) and later presented with partial radiographic responses that, in this analysis, clearly correlate with tumor biomarker responses in patients with advanced PPGL. Further, it should be noted that this unique primary study endpoint (at least a 50% reduction in baseline antihypertensive medication use lasting for ≥6 months) was decided upon in agreement with the FDA in order to establish clinical benefit and it is now further corroborated by the observed substantial biochemical responses.

Biomarker response rates steadily improved during the 12-month efficacy period following the administration of the first dose of HSA I-131 MIBG. The initial improvement, from the 3-month to the 12-month time point, was consistent with receiving both treatment doses (Fig. 2). The continued improvement in the biomarker response following the initial 6-month period likely represents the continued beneficial effects of cytotoxic radiation to the tumor cells, which were slowly progressing through the cell cycle until their eventual apoptosis or necrosis. This is consistent with the finding that 30% of the patients who received two therapeutic doses had confirmed PR that became evident in the long-term follow-up (Pryma *et al.* 2019).

This prospective phase 2 study establishes the benchmarks for PPGL biomarker response to therapy over time. The best overall serum CgA-related tumor marker response rates (CR + PR) were observed to be slightly higher (80%) than total metanephrines (70%). CgA expression correlates with the amount of secretory vesicles in neuroendocrine cells, and serum CgA levels correlate with the release of the contents of these vesicles, including from PPGL tumor cells (Gut *et al.* 2016). The fact that many of the non-primary endpoint responders in our study were nevertheless observed to have an objective tumor response and the majority of patients had a reduction in requirements for antihypertensive medications that lasted less than 6 months, the CgA biomarker may well have captured this non-primary endpoint biochemical response. Importantly, 6 of the 27 patients with elevated CgA that were receiving PPI treatment during the trial either had an SD or PD biomarker response. Given that PPIs are known to increase CgA (Mosli *et al.* 2012), it is possible that the effects of HSA I-131 MIBG therapy on lowering CgA levels were suboptimal in these patients.

While these biomarker responses to HSA I-131 MIBG are robust, this clinical trial was not designed to evaluate the ability of biomarkers to prospectively serve as potential surrogates for tumor response and/or blood pressure response or provide diagnostic performance criteria such as sensitivity or positive predictive value. However, future studies are warranted to assess these questions and/or determine whether a single biochemical response such as CgA, plasma free or 24-h urine fractionated metanephrines could serve to simplify patient biomarker follow-up.

It should also be noted that with a clinical benefit rate that is quite high (>90% when including patients with SD (objective response) who had some degree of regression and substantially improved blood pressure), these findings are quite consistent with the previously published waterfall plot data (Pryma *et al.* 2019). Given the observed correlation between biomarker response and long-lasting reduction in antihypertensive medication demand for these hypersecretory tumors (Table 3), assessment of biomarker response is a reasonable objective therapeutic endpoint. However, the observed correlations were weak to moderate likely due to the variability of the many individual biomarker responses.

The current study is not without its limitations. First, since genetic mutations were not recorded for this study, we were unable to determine whether there were any trends regarding a patient’s genetic background and their biomarker responses. Also, although most patients in the study had hypersecretory tumors at the onset of treatment, all patients had hypertension, suggesting that a small subpopulation may have had increased blood pressure independent of their PPGL. Further, given the challenges of identifying and recruiting patients with this rare disease over a period of many years, no effort could be made to control for the proportion of pheochromocytoma patients relative to paraganglioma patients, or for normal vs elevated baseline biomarker levels for a given assay.

In addition, we were unable to test for plasma methoxytyramine levels since the study was initiated in June 2009, well before its recommended usage for PPGL (Kunz *et al.* 2013). Lastly, longer follow-up data are needed to ascertain the durability of the biomarker response and to determine whether a biomarker response failure may serve as an early indicator of recurrence and/or disease progression, as well as its effect on overall survival.

## Conclusions

The diagnostic value of biomarkers for PPGL has been long recognized. The current study affirms the correlation of biomarker response with objective tumor response by RECIST 1.0 as well as reduction of antihypertensive therapy in patients with advanced PPGL in the setting of a multicenter, prospective phase 2 trial following treatment with HSA I-131 MIBG. Furthermore, these clinical and biochemical responses are durable. Collectively, the findings provide substantial efficacy data for HSA I-131 MIBG for adult and pediatric patients aged 12 years and older with iobenguane scan-positive, unresectable, locally advanced or metastatic PPGL who require systemic therapy.

## Declaration of interest

NS and VAD are employees of Progenics Pharmaceuticals, Inc., a Lantheus company. CJ, BBC, RBN, JSD, LS and DAP served as investigators for this clinical study.

## Funding

Progenics Pharmaceuticals, Inc., a Lantheus company, which has a proprietary commercial interest in AZEDRA® (iobenguane I 131), provided research support for this study.

## Disclaimers

The manuscript and its contents are confidential, intended for journal review purposes only, and not to be further disclosed until published.
